# Structural and Functional Characterization of Cleavage and Inactivation of Human Serine Protease Inhibitors by the Bacterial SPATE Protease EspPα from Enterohemorrhagic *E. coli*

**DOI:** 10.1371/journal.pone.0111363

**Published:** 2014-10-27

**Authors:** André Weiss, Hanna Joerss, Jens Brockmeyer

**Affiliations:** Institute of Food Chemistry, Westfälische Wilhelms-Universität Münster, Münster, Germany; Charité-University Medicine Berlin, Germany

## Abstract

EspPα and EspI are serine protease autotransporters found in enterohemorrhagic *Escherichia coli*. They both belong to the SPATE autotransporter family and are believed to contribute to pathogenicity via proteolytic cleavage and inactivation of different key host proteins during infection. Here, we describe the specific cleavage and functional inactivation of serine protease inhibitors (serpins) by EspPα and compare this activity with the related SPATE EspI. Serpins are structurally related proteins that regulate vital protease cascades, such as blood coagulation and inflammatory host response. For the rapid determination of serpin cleavage sites, we applied direct MALDI-TOF-MS or ESI-FTMS analysis of coincubations of serpins and SPATE proteases and confirmed observed cleavage positions using in-gel-digest of SDS-PAGE-separated degradation products. Activities of both serpin and SPATE protease were assessed in a newly developed photometrical assay using chromogenic peptide substrates. EspPα cleaved the serpins α1-protease inhibitor (α1-PI), α1-antichymotrypsin, angiotensinogen, and α2-antiplasmin. Serpin cleavage led to loss of inhibitory function as demonstrated for α1-PI while EspPα activity was not affected. Notably, EspPα showed pronounced specificity and cleaved procoagulatory serpins such as α2-antiplasmin while the anticoagulatory antithrombin III was not affected. Together with recently published research, this underlines the interference of EspPα with hemostasis or inflammatory responses during infection, while the observed interaction of EspI with serpins is likely to be not physiologically relevant. EspPα-mediated serpin cleavage occurred always in flexible loops, indicating that this structural motif might be required for substrate recognition.

## Introduction

Enterohemorrhagic *Escherichia coli* (EHEC) cause severe diseases in humans worldwide. Shiga toxins are regarded as their main virulence factor. However, EHEC possess various further virulence factors that mediate adherence or interfere with host defense [1], [2]. One of these additional virulence factors is the plasmid-encoded extracellular serine protease EspP which belongs to the serine protease autotransporter of *Enterobacteriaceae* (SPATE) family [3]. Five subtypes of EspP have been described (EspPα-EspPε [4], [5], from which the translocation-competent and proteolytically active subtype EspPα (Uniprot Accession Number: Q7BSW5) is associated with highly virulent strains and isolates from patients with severe disease [4], [6]. EspPα exhibits serine protease activity. In addition to porcine pepsin A and EHEC-Hemolysin [3], [7], EspPα cleaves the human plasma proteins apolipoprotein A-I, the complement factors C3 and C5, and coagulation factor V [3], [8], [9]. EspPα-mediated cleavage of complement factors has been demonstrated to significantly reduce complement activation [9]. In addition, the degradation of factor V has been suggested to interfere with blood coagulation possibly leading to prolonged bleeding during EHEC infection [3].

The *E*. *coli* secreted protease, island-encoded (EspI) is a further member of the SPATE family and is secreted by Shiga toxin-producing *E*. *coli* (STEC) [8]. Notably, EspI has been found in less pathogenic *E*. *coli* serotypes [8], [10], [11]. The physiological function of EspI is yet unknown and to date only two substrates have been identified, namely porcine pepsin A and human apolipoprotein A-I [8].

Serine protease inhibitors (serpins) are structurally closely related proteins which modulate different important protease cascades by irreversible inactivation of serine proteases. They are involved in inflammatory host defense, complement activation, and blood coagulation [12], [13]. Serpins share an exposed reactive center loop (RCL) that serves as a pseudosubstrate for the target protease. Cleavage of the reactive serpin bond initiates a conformational rearrangement of the serpin structure that leads to distortion and inactivation of the target protease by formation of an irreversible covalent serpin-protease complex [14]. α1-protease Inhibitor (α1-PI, Uniprot Accession Number: P01009) is the archetypal member of the serpin family and the most abundant serpin in human plasma. Its main physiological target is neutrophil elastase [15]. α1-antichymotrypsin, (α1-AC, Uniprot Accession Number**:** P01011) which is closely related to α1-PI, [16], [17] mainly inhibits cathepsin G and mast cell chymases [15], [18]. α2-antiplasmin (α2-AP, Uniprot Accession Number: P08697) is the main physiological inhibitor of plasmin and thus influences fibrinolysis following blood coagulation [19], [20]. Antithrombin III (ATIII, Uniprot Accession Number: P01008) inhibits thrombin, FIXa, and FXa - proteases of the blood coagulation pathway - which is considerably faster in the presence of its cofactor heparin [21]–[24]. Angiotensinogen (AGT, Uniprot Accession Number: P01019) is a non-inhibitory serpin that does not target proteases [25]. Via proteolytic processing by renin, AGT releases the vasopressor peptide angiotensin I which is further converted to angiotensin II [26], [27]. An overview of serpin functions and nomenclature is given in Table 1.

**Table 1:** Serpins used in this study.

Serpin	Systematicname	Main Targetproteases	Function	Reference
**α1-Protease** **Inhibitor**	SERPINA1	neutrophilelastase	Protection of tissue duringinflammation, deficiencyresults in emphysema	[15], [56]–[58]
**α1-Antichymotrypsin**	SERPINA3	CathepsinG, mast cell chymases	Deficiency may result inemphysema, possiblecontribution to Alzheimer	[15], [18], [59]–[61]
**Angiotensinogen**	SERPINA8	-	Non-inhibitory, reninsubstrate, release ofangiotensin I	[25], [62]
**α2-Antiplasmin**	SERPINF2	plasmin	Regulation of fibrinolysis	[19], [45]
**Antithrombin III**	SERPINC1	thrombin,FIXa, FXa	Most important inhibitorof the coagulation pathway	[21]–[24]

Given are the systematic serpin name, target proteases, and general function.

Serpins are therefore highly relevant concerning their regulatory function as pseudosubstrates that inactivate serine proteases by formation of serpin-enzyme-complexes. In addition, cleavage of serpins without formation of an inhibitory complex has been described in literature for different metalloproteases. The human matrix metalloproteinase-3, e.g., cleaves α1-AC, α2-AP, and plasminogen activator inhibitor-1 [28], [29] while human matrix metalloproteinase-9 cleaves α1-PI [30]. The bacterial 56-kDa proteinase from *Serratia marcescens* also cleaves α1-PI, α2-AP, ATIII, and C1 esterase inhibitor (C1-INH) [31], [32]. C1-INH is also specifically cleaved by StcE, a metalloprotease found in highly pathogenic EHEC [33]. Surprisingly, interference of StcE with C1-INH also results in enhanced inhibition of complement-mediated lysis irrespective of cleavage of this serpin [34], [35]. Interference with serpin function in the human host during bacterial infection is therefore a further pathogenicity mechanism.

Notably, we describe here the specific cleavage of various serpins from human plasma by the bacterial serine protease EspPα and compare this activity with the related SPATE EspI. Presented data further support the hypothesis that EspPα mediates virulence by interaction with key regulatory proteins of host defense and blood coagulation. In addition, we developed a photometrical assay for the analysis of serpin activity and applied matrix assisted laser desorption ionization-time of flight-mass spectrometry (MALDI-TOF-MS) and electrospray ionisation-fourier transform mass spectrometry (ESI-FTMS) for the direct elucidation of proteolytic cleavage sites.

## Materials and Methods

Pseudonymized residual sample material from voluntary blood donations from the Transfusion medicine of the University Clinics Münster was used. Blood donors approved prior to donation that residual sample material can be used for scientific studies. The Ethics Committee of the Medical Faculty of the University of Münster was informed and approved the study design.

### Proteins

EspPα was purified from clone HB101 (WB4–5k) containing *espP* from *E*. *coli* O157:H7 strain EDL933 [3]. The inactive EspP mutant S263A served as a negative control [36] and EspI was purified in the same way from clone DH5α/pZH4 containing *espI* from *E*. *coli* O91:H^−^ strain 4797/97 [4], [8]. Protein precipitation from culture supernatants was performed as described previously [4]. Briefly, protein pellets were dissolved in 20 mM Tris buffer containing 50 mM NaCl (pH 6.5). Proteins were purified using HiPrep 16/10 DEAE FF, HiTrap Benzamidine FF (HS), and HiPrep 16/60 Sephacryl S-200 HR columns (GE Healthcare) according to the manufactureŕs instructions. Protein preparations were diluted to 1 µg/µL with phosphate buffered saline (PBS, 100 mM NaCl, 4.5 mM KCl, 7.0 mM Na_2_HPO_4_, 3.0 mM KH_2_PO_4_, pH 7.4).

Purified serpins were purchased from Merck Millipore and dissolved according to the manufactureŕs instructions in the following buffers: α1-PI, 30 mM Na_3_PO_4_, 300 mM NaCl, pH 6.5, α1-AC, 20 mM Tris, 250 mM NaCl, 4.5 mM KCl, 7.0 mM Na_2_HPO_4_, 3.0 mM KH_2_PO_4_, pH 7,4, α2-AP, 20 mM Bis-Tris, 200 mM NaCl, pH 6.4, ATIII, 100 mM NaCl, 4.5 mM KCl, 7.0 mM Na_2_HPO_4_, 3.0 mM KH_2_PO_4_, pH 7.4, AGT, 50 mM Na_3_PO_4_, 150 mM NaCl, pH 7.0.

### Plasma fractionation

Plasma samples (fresh frozen plasma, FFP) were stabilized with 17–23% (v/v) citrate-phosphate-dextrose (CPD) and were derived from whole blood donations using standard separation procedures for blood banks.

Plasma was diluted with 20 mM Na_3_PO_4_ buffer (pH 7.0) and depleted using HiTrap Protein A FF and HiTrap Blue HP (GE Healthcare) according to the manufactureŕs instructions. The depleted plasma was further fractionated using HiPrep 16/10 DEAE FF via gradient elution ranging from 100% buffer A (20 mM Tris, 50 mM NaCl, pH 8.0) to 70% buffer B (20 mM Tris, 500 mM NaCl, pH 8.0). The protein fraction eluting from 15–40% buffer B was used for further experiments.

### Cleavage of Substrates

To determine cleavage of substrates by EspPα or EspI, fractionated plasma (25 µg) or serpins (5 µg or 10 µg) were incubated (15 h, 37°C) with 1.5 µg of purified protease in 30 µL PBS buffer. ATIII was incubated in the same way after addition of 25 µg/mL (4.8 units/mL) unfractionated heparin (Merck). Proteins were either separated via sodium dodecyl sulfate-polyacrylamide gel electrophoresis (SDS-PAGE), digested in-gel and analyzed using matrix assisted laser desorption ionization-time of flight-mass spectrometry (MALDI-TOF-MS) or subjected directly to MS analysis.

### SDS-PAGE

After denaturation, proteins were separated on a 7.5% SDS-PAGE gel using a glycine (19.2 mM) containing buffer [37] or on a 13.3% SDS-PAGE gel using a tricine (100 mM) containing buffer [38] and stained with Coomassie Blue.

### In-gel-digestion

In-gel-digestion was performed as described before [39]. Briefly, gel pieces were cut out, proteins were reduced using dithiothreitol (10 mM), alkylated with iodoacetamide (55 mM) and digested (15 h, 37°C) with trypsin (13 ng/µL, Promega). Peptides were extracted and desalted using ZipTip C_18_ Pipette Tips (Merck Millipore) according to the manufactureŕs instructions. Peptides were eluted with 40% acetonitrile (MeCN)/1% formic acid (FA) and 70% MeCN/1% FA (5 µL each) and eluates were combined.

### Mass spectrometric analysis

In-gel-digests or incubation mixtures (0.5 µL) were mixed with 0.5 µL α-cyano-4-hydroxycinnamic acid (Sigma-Aldrich, 10 µg/µL in 50% MeCN/1% trifluoroacetic acid) and 0.5 µL of the mixture were spotted on a MALDI target (MTP 384 target plate ground steel, Bruker). Samples were analyzed using a Bruker autoflex speed in positive mode.

To determine the accurate masses of the largest α2-AP fragment, the incubation mixture was desalted using ZipTip C_18_ Pipette Tips as described before and measured using a Thermo LTQ Orbitrap XL in positive static nanospray mode (sheath gas flow rate 15 arb.u., aux gas flow rate 10 arb.u., sweep gas flow rate 5 arb.u.).

### Determination of EspPα and α1-PI activity

Potential functional consequences of the interaction between α1-PI and EspPα were analyzed by measuring the activities of both proteins after coincubation. To investigate effects of α1-PI on EspPα protease activity, both proteins were incubated together (15 h, 37°C) at equimolar concentrations. Preincubated (15 h, 37°C) EspPα or α1-PI were used as controls. The remaining EspPα protease activity was then determined by the incubation (15 h, 37°C) of an aliquot containing 1 µg EspPα (either preincubated alone or with α1-PI) with 2 mM of the chromogenic peptide substrate Suc-Ala-Ala-Pro-Leu-pNA (Bachem) in 100 µL PBS (pH 7.4) and 5% dimethyl sulfoxide (DMSO). Active EspPα releases para-nitroaniline (pNA) from the peptide which is detected at 405 nm using a FLUOstar Optima plate reader (BMG Labtech). PBS was used as a buffer control.

The effect of EspPα-mediated cleavage on α1-PI serpin activity was determined by coincubation (15 h, 37°C) of α1-PI and EspPα in a molar ratio of 4∶1. Again, incubations (15 h, 37°C) of EspPα or α1-PI alone were used as controls. To assess remaining serpin activity of α1-PI, the coincubation mixture and controls were incubated (5 h, 37°C) with trypsin (Promega) at a molar ratio of α1-PI and trypsin of 4∶1. Active α1-PI inhibits trypsin protease activity. The remaining serpin activity was therefore assessed indirectly by determination of reduced trypsin activity using aliquots of coincubation mixtures and controls containing 0.25 µg trypsin and incubation (2 h, 37°C) with 2 mM of the chromogenic peptide Bz-Arg-pNA (Bachem) in 100 µL PBS (pH 7.4) containing 5% DMSO. Active trypsin releases pNA and absorbance was measured at 405 nm using a FLUOstar Optima plate reader. PBS was used as a buffer control.

## Results and Discussion

### Purification of EspPα and S263A

**Figure 1:**
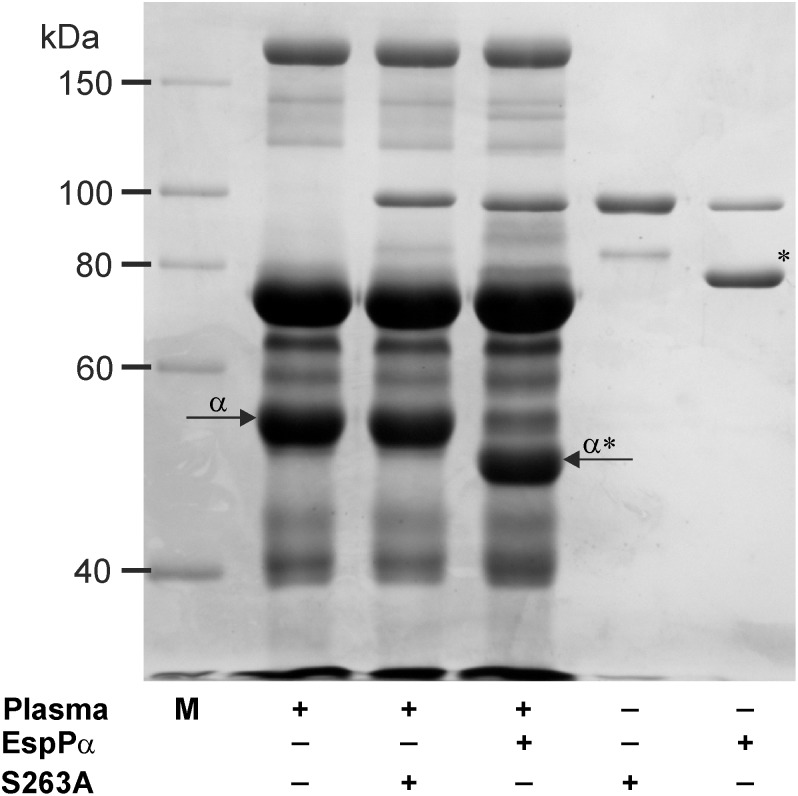
Identification of substrates in plasma. Fractionated plasma (25 µg) was incubated (15 h, 37°C) with EspPα or S263A (1.5 µg) and separated via SDS-PAGE using a glycine buffer. M, molecular weight marker, *, EspPα autodegradation product, α, α1-PI, α*, α1-PI degradation product.

EspPα and the inactive EspPα mutant S263A were purified from culture supernatants using ammonium sulfate precipitation and liquid chromatography. Purity was verified via SDS-PAGE (Fig. 1, lane 5 and 6). EspPα shows a band at ∼104 kDa representing the intact EspPα and a band at ∼80 kDa which was identified by MALDI-TOF-MS as autoproteolysis product. S263A samples showed a pronounced protein band at ∼104 kDa and a weaker band at ∼85 kDa which was identified as a truncated form of S263A. The autoproteolyis product of EspPα remains active even after long term incubation (Figure S1). Proteolytic activity of purified EspPα and the inactive S263A were assessed using a chromogenic oligopeptide substrate. As expected, all EspPα samples were proteolytically active while S263A showed no proteolytic activity (Fig. S1).

### Identification of EspPα substrates in plasma

To identify physiological relevant substrates of EspPα, fractionated plasma was incubated either with EspPα or the EspPα negative control S263A (Fig. 1, lane 3 and 4). Incubation with EspPα resulted in loss of a pronounced 50 kDa band in plasma and the occurrence of a degradation product with a molecular weight of ∼45 kDa in SDS-PAGE. The according protein band was digested in-gel and subjected to MALDI-TOF-MS analysis and unambiguously identified as α1-PI (Aldente score 235.7, sequence coverage 69% to α1-PI (UniProtKB: P01009)).

### EspPα cleaves various serpins

**Figure 2:**
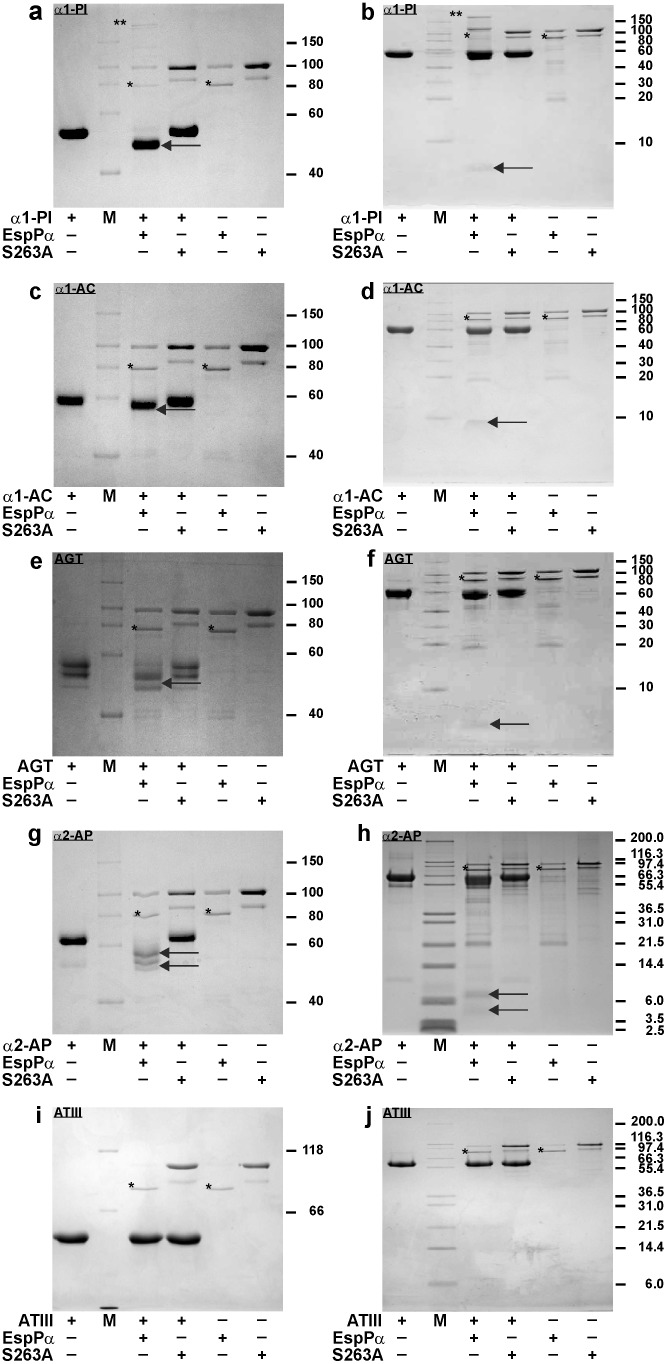
Cleavage of various serpins by EspPα. Serpins (5 µg) were incubated (15 h, 37°C) with EspPα or S263A (1.5 µg). Degradation products were separated via SDS-PAGE using a glycine buffer (a, c, e, g, i) or tricine buffer system (b, d, f, h, j). Proteolytic serpin fragments formed by EspPα are indicated by an arrow. a, b α1-PI is degraded to a large and small fragment (∼45 kDa and ∼4 kDa, respectively), c, d cleavage of α1-AC in two fragments, e, f the AGT band with the highest molecular weight is cleaved in two fragments, g, h large and small fragments (∼55–57 kDa and ∼4–7 kDa) formed by α2-AP cleavage. i, j ATIII is not cleaved by EspPα. Incubation of α1-PI with EspPα leads to a weak formation of an inhibitory enzyme-serpin complex as marked by **. M, molecular weight marker, *, autodegradation product of EspPα.

To determine if further serpins are cleaved by EspPα, different serpins were incubated with EspPα or S263A and cleavage was monitored by SDS-PAGE. EspPα degrades α1-PI, α1-AC, and the non-inhibitory serpin AGT into a large (>40 kDa) and a small (<10 kDa) fragment (Fig. 2 a–f), while incubation of α2-AP leads to several degradation products (Fig. 2 g, h). None of the incubations led to pronounced formation of an inhibitory serpin-enzyme complex. Interestingly, the anticoagulatory serpin ATIII was not degraded by EspPα (Fig. 2i, j).

### Activity of α1-PI and EspPα after incubation

**Figure 3:**
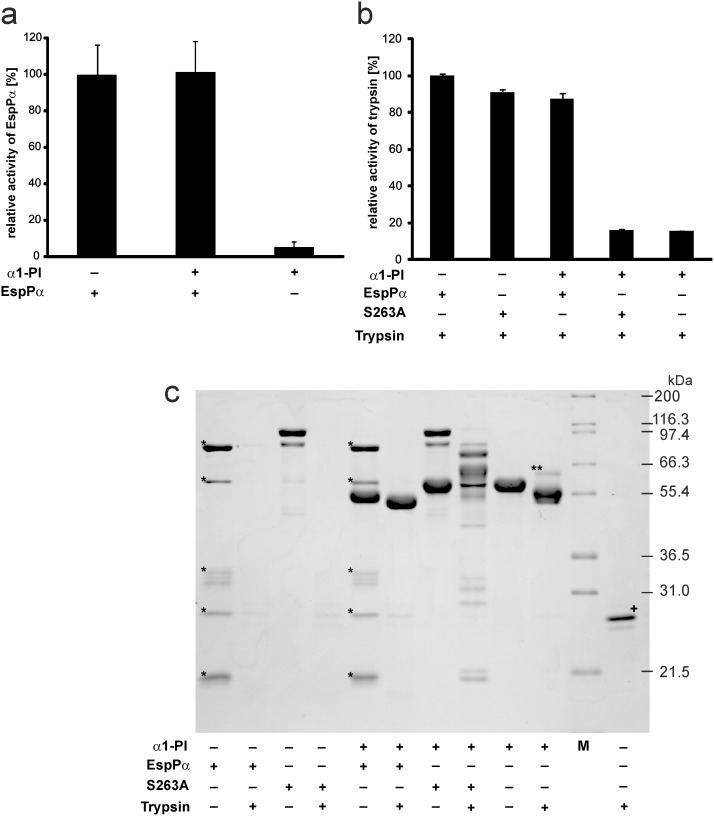
Activity of EspPα and α1-PI after coincubation. a, Determination of EspPα activity. EspPα and α1-PI were preincubated (15 h, 37°C) at equimolar concentrations and remaining activity of EspPα was analyzed by incubation of an aliquot of the mixture with the chromogenic substrate Suc-Ala-Ala-Pro-Leu-pNA. Activity was measured via released *para*-nitroaniline and normalized to EspPα. n = 9 for EspPα and EspPα+α1-PI or n = 6 for α1-PI, respectively. b, α1-PI activity (measured as inhibitory potential on trypsin) after incubation with EspPα. α1-PI and EspPα or S263A were preincubated at a molar ratio of serpin∶enzyme = 4∶1. Remaining inhibitory activity of α1-PI on trypsin was analyzed by incubation at a molar ratio of α1-PI∶trypsin = 4∶1. Trypsin activity was measured via release of *para*-nitroaniline from the chromogenic substrate Bz-Arg-pNA. c, SDS-PAGE analysis of conincubations. α1-PI, EspPα, S263A, and trypsin were incubated as in b) and mixtures were separated via SDS-PAGE (12% SDS-PAGE gel, glycine buffer). M, molecular weight marker, *, EspPα autodegradation product, **, inhibitory complex of α1-PI and trypsin, +, trypsin was directly subjected to SDS-PAGE without incubation.

We next determined the functional consequences of the coincubation of serpin and SPATE protease by use of the *bona fide* serpin α1-PI and EspPα. The remaining EspPα-activity following incubation with α1-PI was assessed in a photometrical assay using the chromogenic EspPα substrate Suc-Ala-Ala-Pro-Leu-pNA. Incubation with α1-PI had no influence on the proteolytic activity of EspPα (Fig. 3a), demonstrating that α1-PI does not target EspPα.

The remaining inhibitory potential of α1-PI following incubation with EspPα was analyzed using trypsin as a serpin target. Although neutrophil elastase is the physiological target for α1-PI, trypsin also forms an irreversible inhibitory complex with the serpin and can therefore be used as an indicator for α1-PI activity [40]. Active α1-PI inhibits the proteolytic activity of trypsin and consequently loss of α1-PI serpin activity results in high proteolytic activity in the assay. Trypsin activity was determined by photometrical detection of the cleavage of the trypsin substrate Bz-Arg-pNA.

Incubation of trypsin with α1-PI or α1-PI preincubated with S263A resulted in nearly complete loss of trypsin activity (Fig. 3b), demonstrating that the employed α1-PI shows high serpin activity and that the inactive EspPα mutant S263A does not affect α1-PI. In contrast, α1-PI preincubated with EspPα did not reduce trypsin activity in the following assay (Fig. 3b). This demonstrates that EspPα-mediated α1-PI cleavage leads to loss of the inhibitory serpin activity. Corresponding results were obtained using SDS-PAGE (Fig. 3c). Incubation of α1-PI with trypsin leads to the formation of a serpin-enzyme-complex (Fig. 3c, lane 10). After incubation with EspPα, α1-PI is not able to form this complex with trypsin. Instead, the large α1-PI fragment is further degraded by trypsin (Fig. 3c, lane 6). EspPα as well as S263A were completely degraded when incubated with trypsin, demonstrating that neither EspPα nor S263A directly interfere with trypsin activity (Fig. 3c, lanes 2 and 4). In addition, α1-PI does not interact with S263A (no serpin enzyme complex) (Fig. 3c, lane 7) but is cleaved by EspPα (Fig. 3c, lane 5). The addition of trypsin to the mixture of α1-PI and S263A led to incomplete degradation and occurrence of several degradation bands in SDS-PAGE. This is due to the fact that degradation of S263A by trypsin and the inhibition of trypsin by α1-PI occur in parallel resulting in only incomplete S263A degradation (Fig. 3c, lane 8).

### EspPα cleaves inside the reactive center loop

**Figure 4:**
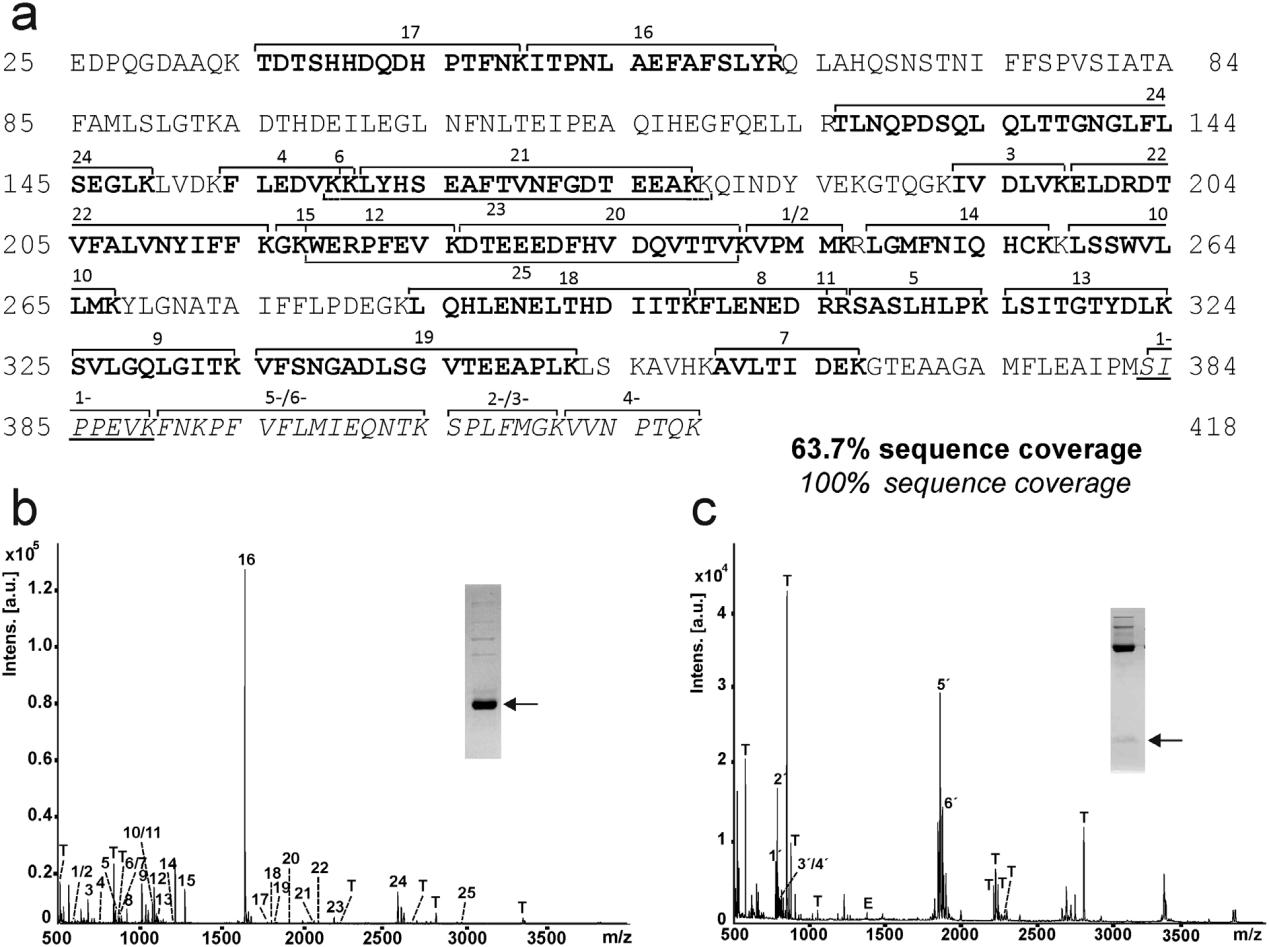
Peptide mapping of EspPα cleavage products of α1-PI. α1-PI fragments were subjected to tryptic in-gel-digest and generated peptides were analyzed via MALDI-TOF-MS. a, Sequence coverage of α1-PI fragments. Peptides of the large fragment are given in bold and numbered 1–25. Peptides of the small fragment are given in italics and numbered 1′-6′. Note the newly formed *N*-terminus of the small fragment (SIPPEVK, underlined). b, MALDI-TOF-MS spectrum of the large fragment of α1-PI. Inset: SDS-PAGE gel, glycine buffer. Fragment used for peptide mapping is marked by arrow. c, MALDI-TOF-MS spectrum of the small fragment of α1-PI. Inset: SDS-PAGE gel, tricine buffer. Fragments used for peptide mapping are marked by arrow. α1-PI peptides are numbered according to a, T, trypsin autoproteolysis products, E, EspPα autoproteolysis products.

The loss of activity of α1-PI but not EspPα is based on cleavage of α1-PI without formation of an inhibitory serpin-enzyme-complex. To further understand how EspPα-mediated cleavage affects the inhibitory function, we determined the cleavage sites in α1-PI and the other serpins included in this study. To this end, large and small fragments of cleaved serpins were separated using SDS-PAGE, in-gel-digested and subjected to MALDI-TOF-MS analysis. Figure 4 shows the peptide mapping of EspPα cleavage products of α1-PI. The large α1-PI fragment consists of the *N*-terminal part of the serpin (Fig. 4a and b), while the *C*-terminal part from residue 383 to 418 forms the small fragment (Fig. 4 a, and c). EspPα cleavage occurs at the active site of the serpin between ^382^Met and ^383^Ser as demonstrated by the occurrence of the non-tryptic peptide 1′(SIPPEVK) and the complete sequence coverage for the small fragment (Fig. 4c). Sequence coverage of degradation products of the other serpins are given in Figure S2.

### Direct MALDI-TOF-MS analysis of small fragments

**Figure 5:**
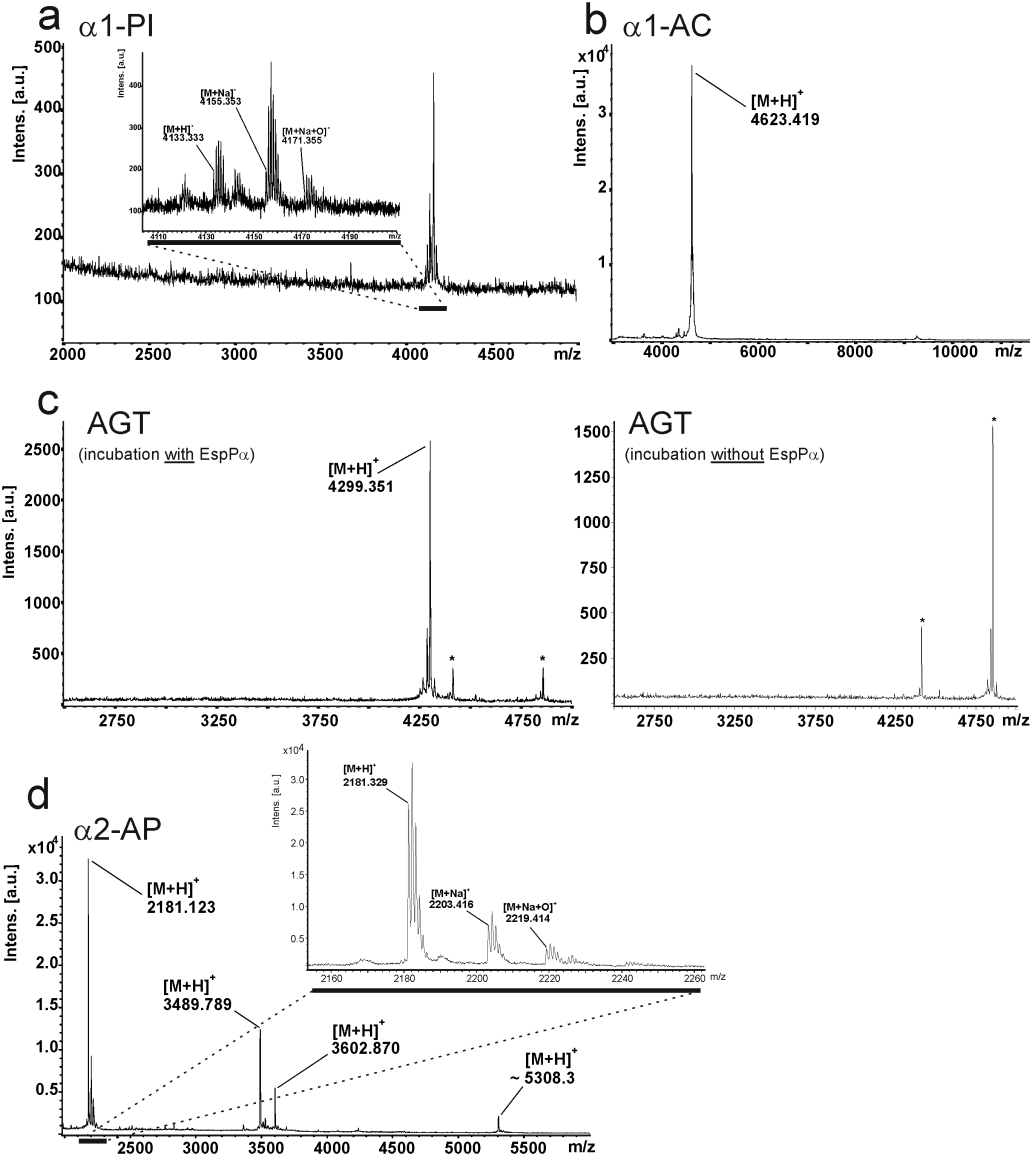
Direct analysis of the small cleavage product of serpins via MALDI-TOF-MS. Serpins were incubated with EspPα and directly analyzed via MALDI-TOF-MS. a, MALDI-TOF-MS spectrum of α1-PI fragment. Inset: Detailed view of the signal representing the small α1-PI fragment. b, MALDI-TOF-MS spectrum of α1-AC. c, MALDI-TOF-MS spectrum of AGT. Left lane: Spectrum after incubation with EspPα, right lane: Spectrum after incubation of AGT without EspPα. *, signals represent non-proteolytic fragments also found after incubation of AGT without EspPα. d, MALDI-TOF-MS spectrum of α2-AP. Inset: Detailed view of the m/z window 2160–2260 representing signals (M H), (M Na), (M Na+O) of the cleavage site in the *N*-terminal extension of α2-AP are exemplarily shown. (M H), proton adduct of small serpin fragment, (M Na), Na adduct of small serpin fragment, (M Na+O), Na adduct oxidized at one methionine residue.

**Figure 6:**
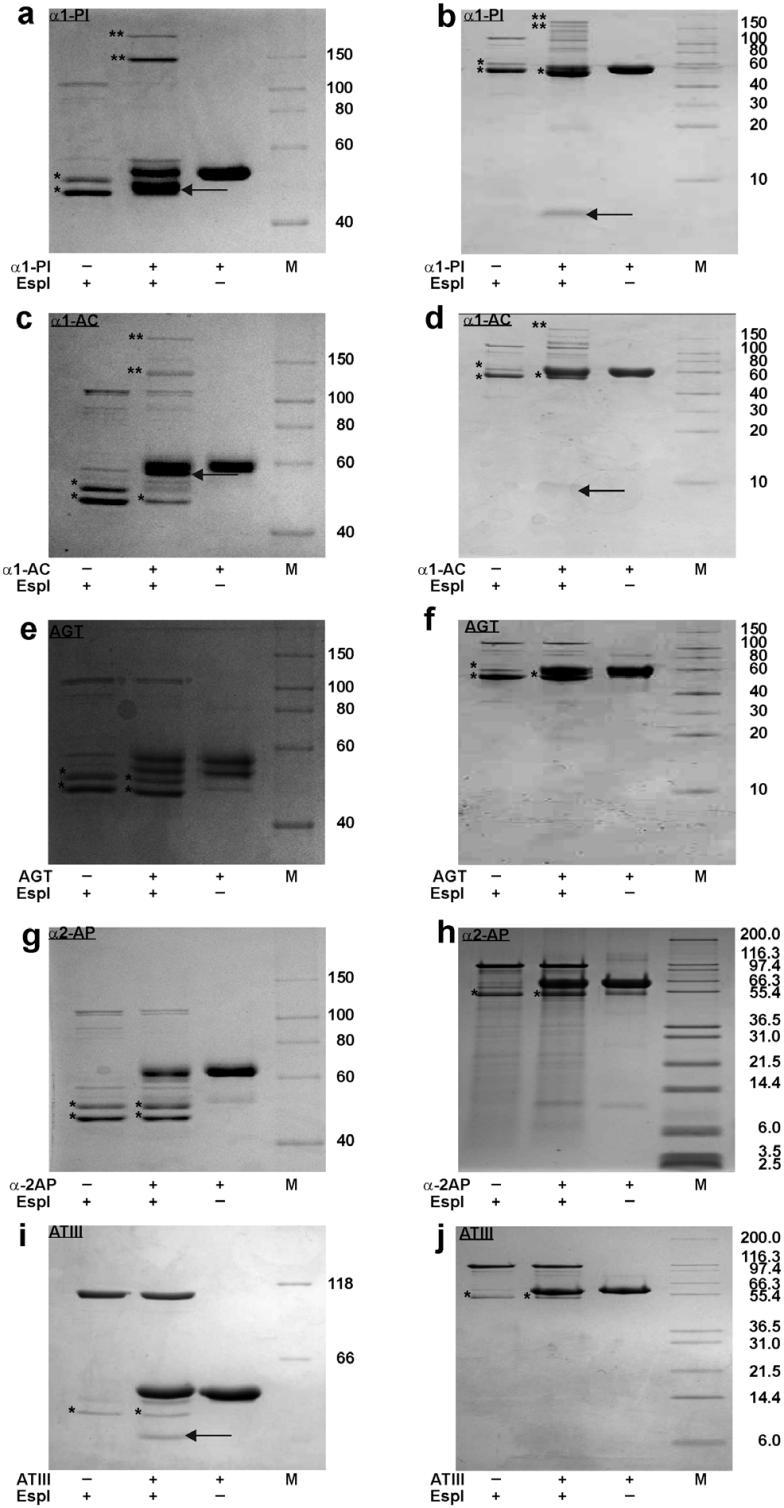
Cleavage of serpins by EspI. Serpins (5 µg) were incubated (15 h, 37°C) with EspI (1.5 µg). Degradation products were separated via SDS-PAGE using a glycine buffer (a, c, e, g, i) or a tricine buffer (b, d, f, h, j). a, b α1-PI is cleaved into two fragments (∼45 kDa and ∼4 kDa), c, d α1-AC is cleaved into two fragments, e, f AGT is not cleaved by EspI, g, h α2-AP is not cleaved by EspI, i, j ATIII is cleaved only with very low efficiency. Note the formation of inhibitory serpin-enzyme-complexes after incubation with α1-PI and α1-AC. M, molecular weight marker, *, autodegradation product of EspI, **, inhibitory serpin-EspI-complex. Serpin fragments are indicated by an arrow.

Not all cleavage sites can be identified via in-gel-digest. Tryptic peptides might be too small when cleavage occurs close to lysine or arginine residues or when several cleavage sites are in close proximity to each other. As all small fragments formed by EspPα-cleavage show a molecular weight below 10 kDa, we applied direct MALDI-TOF-MS analysis to determine the exact mass of the small serpin fragments to elucidate and confirm cleavage sites (Fig. 5). For the small α1-PI fragment we observed a signal for the proton adduct of the α1-PI sequence ^383^Ser-^418^Lys (m/z 4133.333) confirming the cleavage site determined via in-gel-digest. In addition, signals representing the Na^+^ adduct and the oxidized Na^+^ adduct of the according α1-PI fragment sequence were observed (Fig. 5a). α1-AC shows a similar spectrum with a pronounced signal at m/z 4623.419 demonstrating cleavage C-terminal of ^383^Leu at the reactive bond (Fig. 6b), which is in good accordance with data from in-gel-digest (Figure S2). For AGT, we already observed three bands in SDS-PAGE (intact AGT and two non-proteolytic fragments) when incubated without protease (Fig. 2e). Accordingly, signals of two small AGT fragments were observed in MALDI-TOF-MS (Fig. 5c, right lane). Incubation with EspPα led to degradation of intact AGT and occurrence of the corresponding small fragment in MALDI-TOF-MS (Fig. 2e and Fig. 5c, left lane). For α2-AP, proteolytic cleavage into several fragments is observed in SDS-PAGE (see Fig. 2g and 2h and Fig. 5d) after incubation with EspPα. Four distinct signals are seen in the MS spectrum indicating 4 cleavage sites. As the resolution for the signal at m/z 5308.3 is too low to determine the monoisotopic mass, we measured this sample in addition via nanospray-ESI-FTMS. Table 2 summarizes EspPα cleavage sites and their positions within the respective serpin. Measurement of α2-AP after incubation with EspPα via nanospray ESI-FTMS is described in Table 3.

**Table 2:** Serpin cleavage sites determined by MALDI-TOF-MS.

Serpin	m/z determined	Theoretical mass	Deviation (ppm)	Sequence	Position
**α1-PI**	4133.333	4133.234	+24	^380^IPM-SIP^385^	Reactive bond
**α1-AC**	4623.419	4623.495	−16	^381^TLL-SAL^386^	Reactive bond
**AGT**	4299.351	4299.293	+14	^444^QQL-NKP^449^	Reactive center loop
**α2-AP**	2181.123	2181.097	+12	^45^SPL-TLL^50^	*N*-terminal extension
**α2-AP**	3489.789	3489.788	<1	^458^QSL-KGF^463^	*C*-terminal extension
**α2-AP**	3602.870	3602.872	−1	^457^LQS-LKG^462^	*C*-terminal extension
**α2-AP**	5308.3 (average)	5307.9 (average)	+75	^442^REL-KEQ^447^	*C*-terminal extension

Given are masses determined by MALDI-TOF-MS directly after incubation of serpin with EspPα, theoretical masses, mass deviation, according sequence, and position inside the serpin sequence. Numeration is according to the serpin precursor.

**Table 3:** α2-AP cleavage site determined by ESI-FTMS.

m/z determined	m/z theoretical	Charge state (z)	Deviation (ppm)	Sequence	Position
**884.9507**	884.9512	6	−1	^442^REL-KEQ^447^	*C*-terminal extension
**758.6725**	758.6736	7	−1	^442^REL-KEQ^447^	*C*-terminal extension
**663.9624**	663.9653	8	−4	^442^REL-KEQ^447^	*C*-terminal extension

Given are masses of the large α2-AP fragment as determined by nanospray ESI-FTMS.

α1-PI and α1-AC are cleaved at their reactive bonds (position of reactive sites are described in [41], [42]), leading to loss of serpin function. In both molecules the reactive bonds are exposed in the RCL and serve as pseudosubstrates for the targeted proteases. In case of EspPα, the serpins are not able to form a stable inhibitor-enzyme-complex and therefore release the intact EspPα after cleavage. Although AGT as non-inhibitory serpin does not contain a reactive bond, it is structurally closely related to the other serpins and is also cleaved in the RCL, indicating that a reactive bond is not necessary for EspPα-mediated serpin degradation. This is further underlined for α2-AP, which is cleaved at four positions outside the RCL (for RCL position see [43]). Cleavage sites are located at the *N*- and *C*-terminal extensions 25 aa downstream the *N*-terminus and 46, 31, and 30 aa upstream the *C*-terminus (see Table 2). Intriguingly, both the *N*- and *C*-terminal extensions are vital for the functional relevant binding of α2-AP to other proteins [19], [44], [45].

### Cleavage of serpins by EspI

Purified EspI samples showed a protein band at ∼110 kDa (intact EspI) as well as two EspI autoproteolysis products at ∼50 and 45 kDa, respectively. Similar to EspPα, autoproteolysis products remain active. Serpins were incubated with purified EspI in the same way as described for EspPα. Incubation of α1-PI and α1-AC with EspI led to degradation of these serpins. Notably, EspI also forms a pronounced inhibitory complex with both protease inhibitors resulting in only incomplete serpin degradation (Fig. 6 a-d). In contrast to EspPα, EspI does not cleave α2-AP and AGT (Fig. 6e–h). Cleavage of ATIII occurred only with very low efficiency (Fig. 6i) and might not be relevant under physiological conditions.

To determine the cleavage sites of α1-PI and α1-AC, we subjected incubation mixtures of serpins and EspI to direct MALDI-TOF-MS analysis. Serpin cleavage occurred at the reactive bond leading to signals at m/z 4155.400 (α1-PI, 20 ppm deviation according to calculated m/z) and 4623.509 (α1-AC, 19 ppm deviation according to calculated m/z), respectively (data not shown).

## Conclusions

EspPα is an EHEC virulence factor that belongs to the SPATE family. As suggested for SPATEs in general, EspPα most likely mediates its virulence via cleavage and inactivation of host proteins. Here, we present a method for the rapid determination of EspPα-mediated cleavage sites in various human plasma serpins via MALDI-TOF-MS as well as a photometrical assay to analyze serpin functionality after proteolytic cleavage. Concerning the functional consequences, degradation of α2-AP might lead to bleeding disorders. This serpin is the primary physiological inhibitor of plasmin and deficiency has been shown to result in uncontrolled fibrinolysis and severe hemorrhagic complication [44], [45]. α2-AP harbors a 42 aa *N*-terminal and a 55 aa *C*-terminal extension [19], [46]. While the *N*-terminal extension is cross-linked to fibrin, the very *C*-terminal ^491^Lys residue mediates binding to plasmin [47]. EspPα cleaves between ^47^Leu and ^48^Thr releasing part of the *N*-terminal extension and at three different sites inside the *C*-terminal extension leading to release of a polypeptide containing ^491^Lys. Together, this most likely leads to loss of function of α2-AP. The role of α1-PI in thrombosis is not well understood. However, α1-PI is able to inhibit activated protein C. In pediatric ischemic stroke patients elevated levels of α1-PI have been found and were discussed to contribute to this thrombotic disease in children [48], [49]. ATIII is the main anticoagulatory serpin. Although it is able to interfere with virtually all proteolytic coagulation factors, its main targets are thrombin, FIXa, and FXa. Intriguingly, it is the only serpin in this study that is not cleaved by EspPα. Despite the structural similarity of serpins, EspPα specifically cleaves only selected serpins. More specific, procoagulatory serpins such α2-AP and α1-PI are efficiently degraded while the anticoagulatory ATIII is not affected at all. Together with data demonstrating that EspPα cleaves coagulation factor V [3], this underlines the hypothesis that interference with blood coagulation (and possibly also inflammatory host responses) [50] might be one of the major functions of EspPα which might contribute to formation of hemorrhages observed during EHEC infection.

**Figure 7:**
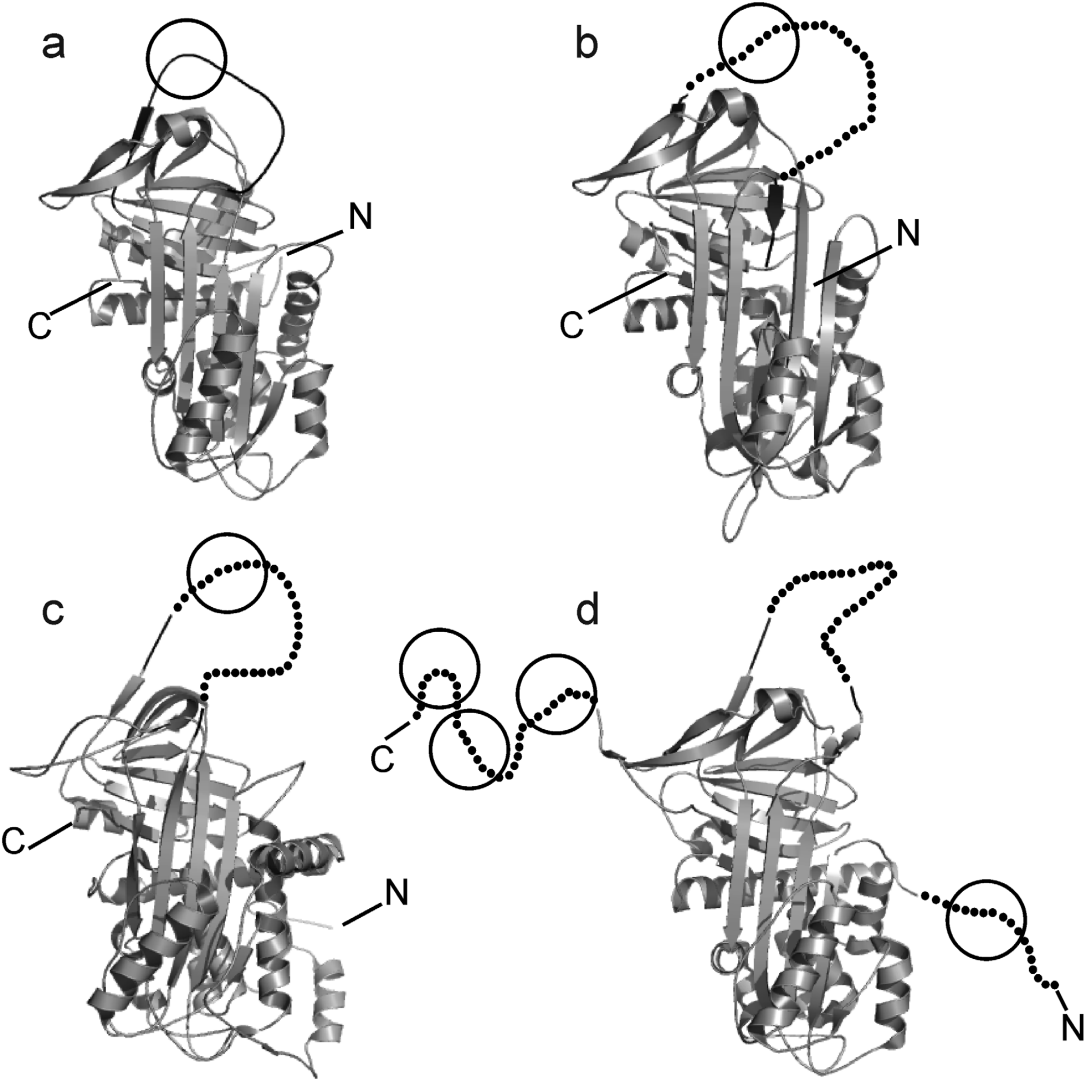
Crystal structures of serpins cleaved by EspPα. Serpins are shown as cartoons. RCL is indicated in black, approximate cleavage sites are encircled. Non-resolved parts of the crystal structures are indicated by dots (c, RCL of AGT, d, RCL of α2-AP and the *N*- and *C*-terminal extension of α2-AP). a, human α1-PI, b, cleaved human α1-AC, the RCL is indicated by dots, c, human angiotensinogen, d, murine truncated α2-AP_Δ43_, the *N*-terminal extension of native α2-AP is indicated by dots.

Having a closer look at EspPα cleavage sites, it is notable that more than 70% (5 of 7) of cleavage sites identified in this study occur after Leu. This is in good accordance to already reported EspPα cleavage sites [3], [9], [7], [51], indicating that substrate cleavage is most favorable *C*-terminal to Leu. In α2-AP, cleavage also occurs after ^459^Ser. This residue, however, is positioned next to ^460^Leu after which EspPα cleaves, too. The second non-Leu cleavage site is C-terminal to ^382^Met in α1-PI. The ^382^Met-^383^Ser bond, however, is the reactive bond exposed in the RCL and required to react with target proteases. Similarly, α1-AC is cleaved at the reactive bond that consists of a Leu-Ser motif which is also located in the exposed RCL. Cleavage of the non-inhibitory AGT shows that a reactive bond is not strictly required for substrate recognition by EspPα but cleavage also occurs inside the corresponding reactive center loop. In contrast, α2-AP is not cleaved in the RCL but inside the *N*- and *C*-terminal extensions which are vital for α2-AP functionality. Though the crystal structure of α2-AP has only been solved for a *N*-terminally truncated murine form, it seems that the *C*-terminal extension consists of a flexible loop because it could not be modeled into electron density maps [52]. Perhaps, this structural flexibility seen in the reactive center loops and in the *C*-terminal extension of α2-AP is required for substrate recognition by EspPα. Figure 7 shows crystal structures of the serpins that are cleaved by EspPα [52]–[55].

EspI shows significant differences in substrate specificity compared to EspPα. α1-PI and α1-AC are also cleaved at their reactive bonds which should lead to loss of function of these serpins. However, serpin cleavage and release of the protease is not complete for EspI, most probably due to the pronounced formation of an inhibitory serpin-enzyme-complex of EspI with α1-PI and α1-AC. In contrast, EspPα completely degrades both serpins and forms only small amounts of the inhibitory complex only with α1-PI which does not significantly reduce EspPα activity. In addition, AGT and α2-AP, which are degraded by EspPα at positions other than the reactive bond, are not degraded by EspI. Concerning the functional differences of both SPATE proteases, EspPα is able to cleave serpins specifically within accessible loop structures and is notably not inhibited by the analyzed serpins, while EspI is only able to interact with the reactive bond of α1-PI and α1-AC. The latter interactions show equilibria between EspI inhibition and serpin degradation. Taking into account the high amounts of serpins such as α1-PI in plasma, EspI activity might be strongly reduced in this milieu in vivo, while serpin degradation and inactivation might be a relevant function of EspPα also during infection.

In summary, we established a rapid method to determine cleavage sites of small proteolytic fragments via MALDI-TOF-MS. Functional implications have been investigated in a newly developed photometrical assay using chromogenic peptide substrates. EspPα degrades and thereby inactivates different plasma serpins which, in case of α2-AP, might lead to bleeding disorders or in case of α1-PI and α1-AC might interfere with the acute phase reaction during inflammatory host response. Cleavage occurs in flexible regions most favorable *C*-terminal to Leu. Comparison of EspPα and EspI indicate different functions of this SPATE also in vivo.

## Supporting Information

Figure S1 **Activity of EspPα and S263A.** a, Determination of EspPα and S263A activity directly after purification. EspPα or S263A was incubated (15 h, 37°C) with the chromogenic substrate Suc-Ala-Ala-Pro-Leu-pNA. Activity was measured via released *para*-nitroaniline and normalized to EspPα. PBS was used as control. n = 2, b, Determination of EspPα activity after preincubation. Purified EspPα was preincubated for 15 h at 37°C resulting in the formation of autoproteolysis products (see Fig. 3c, lane1). To assess remaining proteolytic activity of autoproteolysis products the preincubated sample was incubated with the chromogenic substrate Suc-Ala-Ala-Pro-Leu-pNA (15 h, 37°C). Again, activity was measured via released *para*-nitroaniline and normalized to EspPα. PBS was used as control. n = 2.
(TIFF)

Figure S2 **Peptide mapping of EspPα cleavage products of the serpins.** Serpin fragments were subjected to in-gel-digest and analyzed via MALDI-TOF-MS. Peptides of the large fragment are given in bold. Peptides of the small fragments are given in italics, a, sequence coverage of α1-AC fragments, b, sequence coverage of AGT, c, sequence coverage of α2-AP. Note that in the small fragments of AGT and α2-AP no serpin peptides were found.
(TIF)
