# Supplementary figures and images for: Structural and Functional Characterization of Cleavage and Inactivation of Human Serine Protease Inhibitors by the Bacterial SPATE Protease EspPα from Enterohemorrhagic E. coli
Source: PLoS One. 2014 Oct 27;9(10):e111363. doi: 10.1371/journal.pone.0111363 (PMC4210187; doi:10.1371/journal.pone.0111363)

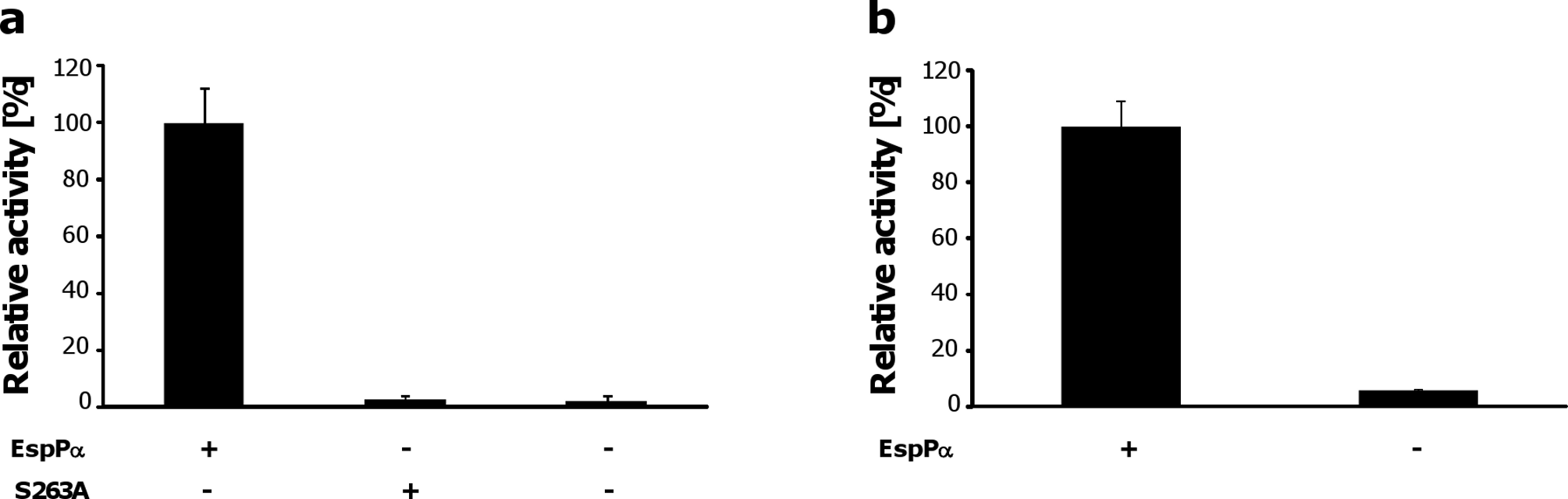

Supplement: Figure S1 — Activity of EspPα and S263A. a, Determination of EspPα and S263A activity directly after purification. EspPα or S263A was incubated (15 h, 37°C) with the chromogenic substrate Suc-Ala-Ala-Pro-Leu-pNA. Activity was measured via released para-nitroaniline and normalized to EspPα. PBS was used as control. n = 2, b, Determination of EspPα activity after preincubation. Purified EspPα was preincubated for 15 h at 37°C resulting in the formation of autoproteolysis products (see Fig. 3c, lane1). To assess remaining proteolytic activity of autoproteolysis products the preincubated sample was incubated with the chromogenic substrate Suc-Ala-Ala-Pro-Leu-pNA (15 h, 37°C). Again, activity was measured via released para-nitroaniline and normalized to EspPα. PBS was used as control. n = 2. (TIFF) [file pone.0111363.s001.tiff]

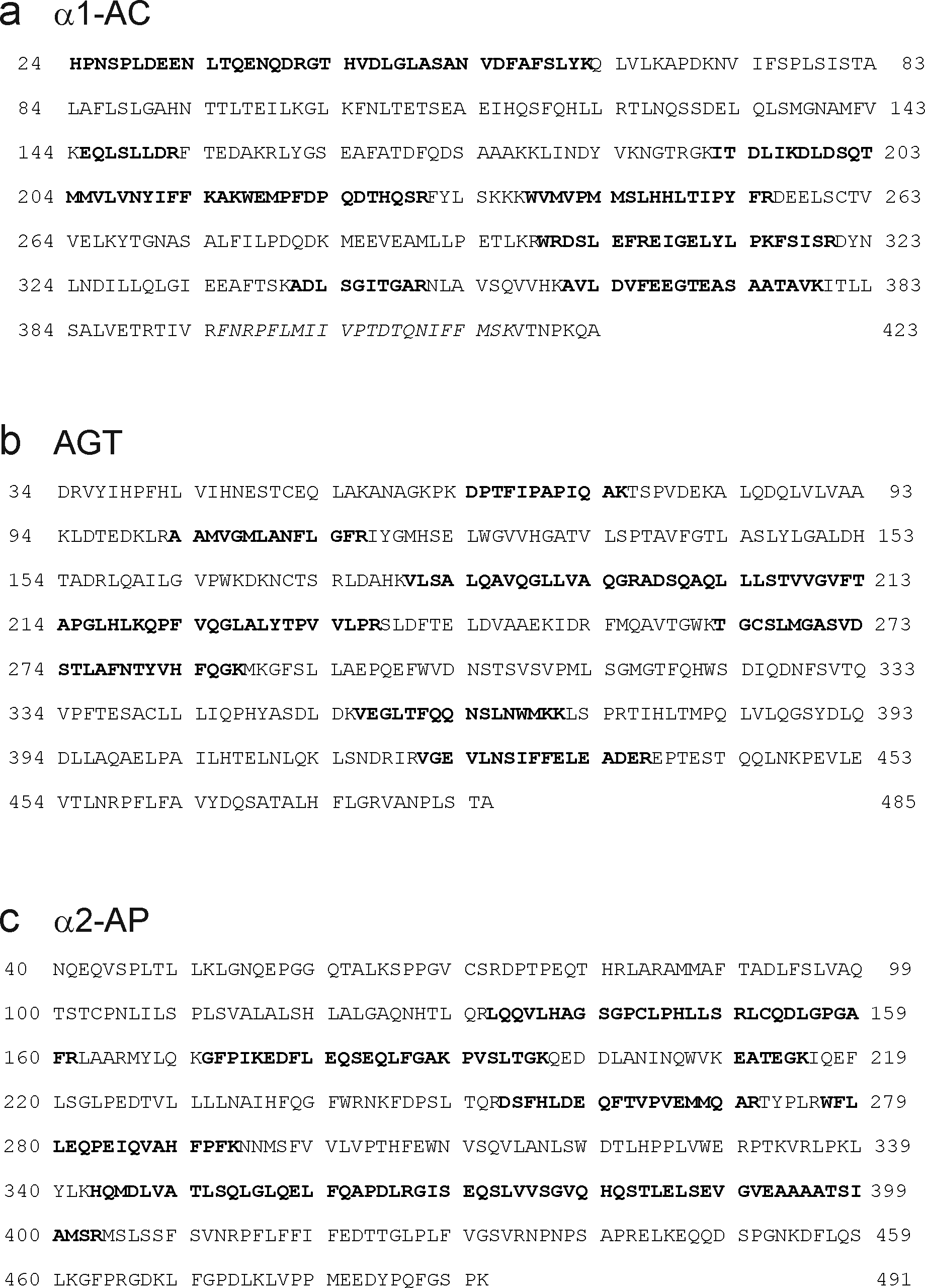

Supplement: Figure S2 — Peptide mapping of EspPα cleavage products of the serpins. Serpin fragments were subjected to in-gel-digest and analyzed via MALDI-TOF-MS. Peptides of the large fragment are given in bold. Peptides of the small fragments are given in italics, a, sequence coverage of α1-AC fragments, b, sequence coverage of AGT, c, sequence coverage of α2-AP. Note that in the small fragments of AGT and α2-AP no serpin peptides were found. (TIF) [file pone.0111363.s002.tif]
